# Risk factors for Mycobacterium tuberculosis infection in 2–4 year olds in a rural HIV-prevalent setting

**DOI:** 10.5588/ijtld.15.0672

**Published:** 2016-03

**Authors:** P. Y. Khan, J. R. Glynn, K. L. Fielding, T. Mzembe, D. Mulawa, R. Chiumya, P. E. M. Fine, O. Koole, K. Kranzer, A. C. Crampin

**Affiliations:** * Department of Infectious Disease Epidemiology, London School of Hygiene & Tropical Medicine, London, UK;; † Karonga Prevention Study, Chilumba, Malawi; ‡ National and Supranational Mycobacterium Reference Laboratory, Forschungszentrum Borstel, Borstel, Germany

**Keywords:** *M. tuberculosis* infection, risk factors, children, community, household, HIV

## Abstract

BACKGROUND: Mycobacterium tuberculosis infection in children acts as a sentinel for infectious tuberculosis.

OBJECTIVE: To assess risk factors associated with tuberculous infection in pre-school children.

METHOD: We conducted a population-wide tuberculin skin test (TST) survey from January to December 2012 in Malawi. All children aged 2–4 years residing in a demographic surveillance area were eligible. Detailed demographic data, including adult human immunodeficiency virus (HIV) status, and clinical and sociodemographic data on all diagnosed tuberculosis (TB) patients were available.

RESULTS: The prevalence of M. tuberculosis infection was 1.1% using a TST induration cut-off of 15 mm (estimated annual risk of infection of 0.3%). The main identifiable risk factors were maternal HIV infection at birth (adjusted OR [aOR] 3.6, 95%CI 1.1–12.2), having three or more adult members in the household over a lifetime (aOR 2.4, 95%CI 1.2–4.8) and living in close proximity to a known case of infectious TB (aOR 1.6, 95%CI 1.1–2.4), modelled as a linear variable across categories (>200 m, 100–200 m, <100 m, within household). Less than 20% of the infected children lived within 200 m of a known diagnosed case.

CONCLUSION: Household and community risk factors identified do not explain the majority of M. tuberculosis infections in children in our setting.

## RESUME

CONTEXTE : L'infection à Mycobacterium tuberculosis chez l'enfant se comporte comme une sentinelle de la tuberculose (TB) contagieuse. Le but de l'étude a été d'évaluer les facteurs de risque associés à l'infection chez des enfants d'âge préscolaire.

MÉTHODE : Nous avons réalisé une enquête par test cutané à la tuberculine (TST) dans toute la population de janvier à décembre 2012 au Malawi. Tous les enfants âgés de 2 à 4 ans résidant dans une zone de surveillance démographique ont étééligibles. Des données démographiques détaillées, notamment le statut par rapport au virus de l'immunodéficience humaine (VIH) de l'adulte et les données cliniques et sociodémographiques concernant tous les patients ayant eu un diagnostic de TB ont été disponibles.

RÉSULTATS : La prévalence de l'infection à M. tuberculosis a été de 1,1% en utilisant comme seuil une induration du TST de 15 mm (risque annuel estimé d'infection de 0,3%). Les principaux facteurs de risque identifiables ont été l'infection à VIH maternelle à la naissance (OR ajusté [ORa] 3,6 ; IC95% 1,1–12,2), la présence de ⩾3 adultes dans le même foyer pendant toute leur vie (ORa 2.4 ; IC95% 1,2–4,8) et le fait de vivre à proximité d'un cas de TB contagieuse connu (ORa 1,6 ; IC95% 1,1–2,4), modélisé comme une variable linéaire à travers les différentes catégories (>200 m, 100–200 m, <100 m, au sein du foyer). Moins de 20% des enfants infectés vivaient dans un rayon de 200 m d'un cas diagnostiqué connu.

CONCLUSION : Les facteurs de risque identifiés au niveau des foyers et des communautés n'expliquent pas la majorité des infections à M. tuberculosis des enfants dans notre contexte.

## RESUMEN

MARCO DE REFERENCIA: La infección por Mycobacterium tuberculosis en los niños cumple una función centinela de la tuberculosis (TB) contagiosa. El presente estudio tuvo por objeto evaluar los factores de riesgo de contraer la infección en los niños de edad prescolar.

MÉTODO: Se llevó a cabo una encuesta tuberculínica (TST) a escala poblacional de enero a diciembre del 2012 en Malawi. Todos los niños de edad de 2–4 años residentes en una zona de vigilancia demográfica eran aptos para participar en la encuesta. Todos los pacientes con diagnóstico de TB contaban con datos demográficos exhaustivos, incluida la situación frente al virus de la inmunodeficiencia humana (VIH), los datos clínicos y sociodemográficos.

RESULTADOS: La prevalencia de infección tuberculosa fue 1,1%, al utilizar un umbral discriminatorio de 15 mm de induración (un riesgo anual de infección de 0,3%). Los principales factores de riesgo de contraer la infección que se detectaron fueron la infección materna por el VIH en el momento del parto (OR ajustado [ORa] 3,6; IC95% 1,1–12,2); haber cohabitado con ⩾3 adultos miembros del hogar durante toda la vida (ORa 2,4; IC95% 1,2–4,8); y el hecho de haber vivido próximo a un caso conocido de TB contagiosa (ORa 1,6; IC95% 1,1–2,4), modelado como una variable lineal en todas las categorías (a >200 m, de 100 m a 200 m, <100 m y en el domicilio). Menos de 20% de los niños infectados había vivido a <200 m de un caso diagnosticado conocido.

CONCLUSIÓN: Los factores de riesgo domiciliarios y comunitarios de contraer la infección tuberculosa que se detectaron no explican la mayor parte de estas infecciones en los niños de este entorno.

MYCOBACTERIUM TUBERCULOSIS infection in children aged <5 years indicates recent transmission and acts as a sentinel for infectious (typically adult) tuberculosis (TB).^1–4^ Accurately identifying M. tuberculosis infection in children can highlight recent failures in control measures in the community,^1^ and identify a population at high risk of progression to active disease.^5^ Untreated M. tuberculosis infection in children will ultimately form the reservoir from which future infectious cases will arise.^2^,^6^ Understanding and managing this reservoir is critical to achieving the ambitious Stop TB Partnership target of TB elimination by 2050.^7^

Most M. tuberculosis transmission to young children is thought to result from household contact.^1^ While household contacts of TB cases are at high risk for infection and disease,^8^ in endemic areas most adult TB appears to occur from transmission outside the household.^9–11^ This may also be true for paediatric TB, but there is little evidence to date,^12^ and results are conflicting. Wide variations in estimates of the proportion of M. tuberculosis infection in children attributable to contact with infectious adults within the household have been reported, ranging from 75% in Cape Town^13^ to <1% in a peri-urban shanty town in Peru.^14^ Differences in settings, such as community human immunodeficiency virus (HIV) and TB prevalence, population density and housing, and differences in age groups, with varying degrees of susceptibility to infection^15^ and disparate social contact patterns,^16^ contribute to differences in the estimates reported.

Within the context of a well-implemented TB control programme, we aimed to assess household and community risk factors associated with M. tuberculosis infection in pre-school children to help elucidate factors driving M. tuberculosis transmission that are not being addressed by current prevention strategies.

## STUDY POPULATION AND METHODS

### Study setting

Karonga District, northern Malawi, is predominantly rural, with an adult HIV prevalence of around 9%, and an incidence of new smear-positive TB of 87 per 100 000 adults per year.^17^ Bacille Calmette-Guérin (BCG) vaccination is administered to children on first health system contact (usually birth) as part of the Expanded Programme on Immunisation.

The present study was conducted in the Karonga demographic surveillance site (DSS), with a population of approximately 36 000, which has been described in detail elsewhere.^18^ The entire population is seen annually for re-census and related surveys, and 97% of all births and 99% of all deaths are reported by key informants.^18^ Key informants are responsible community individuals who are trained to report vital events at a monthly meeting at which they are given a nominal sum (of about US$3) as compensation.^19^ The demographic data include detailed information on family relationships, household socio-economic status, global positioning system (GPS) co-ordinates, dwelling structure, screening for chronic cough (⩾2 weeks among those seen) and vaccination history of children aged ⩽5 years. Adult HIV status (age ⩾15 years) was collected in HIV prevalence surveys from 2007 to 2011 and other studies.

### Participants

We conducted a population-wide tuberculin skin test (TST) survey in 2012 among all children aged 2–4 years residing within the DSS at the time of household recruitment. Children whose mothers were known to be HIV-positive at the time of delivery were defined as HIV-exposed, while children whose mothers were HIV-negative after or up to 1 year before their birth were defined as non-HIV-exposed.

A composite dwelling index was generated based on the building materials used and the presence or absence of glass windows.^20^ A composite asset index was constructed by summing the ownership of household items, weighted by the monetary value of each item, to create an asset score.^21^ The lowest 10–15% scores were coded as ‘1’, the highest 15% as ‘4’ and the middle groups were divided into ‘2’ and ‘3’ to create the respective socio-economic indices.

### Diagnosed tuberculosis cases

The Karonga Prevention Study (KPS) collaborates with the district National Tuberculosis Programme to support core TB prevention and care activities. Bacteriological (including smear and culture status), demographic (including GPS co-ordinates of TB case household/s) and clinical (including HIV status) data were available from an ongoing prospective cohort of all patients starting treatment for TB in the district.^17^ A total of 108 adult (aged ⩾15 years) residents of the DSS were diagnosed with smear-positive pulmonary TB during 2007–2012.

The average annual smear-positive notification rate for each predefined residential area of approximately 450 households was calculated as the number of smear-positive TB cases reported per year per 100 000 population for the period 2007–2012.^22^ Distance to the nearest smear-positive pulmonary TB case was calculated using ArcGIS 10^®^ software (Environmental Systems Research Institute, Redlands, CA, USA). A categorical variable was generated taking into account the variation in distance to the closest neighbour within the DSS. Preparatory work with parents identified that children aged <5 years did not generally venture further than ‘calling’ distance from home, which was estimated to be about 200 m. The variable was therefore categorised to include household contact, neighbourhood contact (resident within 100 m; resident within 100–200 m) and children who lived >200 m from a known smear-positive TB case (baseline category).

### Study procedures

Field staff were trained in TST placement and reading according to standard international guidelines,^23^ using 2 international units of RT23 (Statens Serum Institute, Copenhagen, Denmark) and measuring the induration 48–72 h later. Periodic blinded comparison readings were done against the same reference reader to ensure consistency of TST reading.

Children with TST ⩾10 mm were assessed for TB-related symptoms by field staff, and the results were recorded in the child's health passport. Any child with symptoms suggestive of TB (fever, weight loss, failure to thrive, night sweats or cough) was reviewed by a clinician and referred to the district hospital. The HIV status of the children was not determined unless clinically indicated at the hospital. All children with TST ⩾15 mm were commenced on 6-month isoniazid preventive therapy (IPT) (10 mg/kg once daily) after active disease had been excluded.

### Case definition

A positive TST was defined as an induration of ⩾15 mm, based on a previous mixture analysis of a tuberculin survey conducted in Karonga in 1980–1984 to estimate infection prevalence where exposure to environmental mycobacteria is common.^24^ To check the suitability of this cut-off, the analysis was repeated in the new data set.

### Statistical analysis

Mixture analysis of tuberculin data was based on implementation of the Expectation Maximization (EM) algorithm in *R* (R Foundation for Statistical Computing, Vienna, Austria) by fitting a two-component model to the observed profiles of the non-zero indurations,^24^,^25^

Annual risk of infection was calculated using the formula:^2^

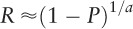
where *R* = annual risk of infection, *P* = proportion of children with ‘positive’ TST and *a* = the mean age of the children.

A random effects logistic regression model taking into account clustering within the residential area was used to assess the relationship between risk factors and TST positivity. The likelihood ratio test was used to assess the overall significance of risk factors, tests for trend and departures from linearity, unless otherwise specified. To prevent over-parameterisation,^26^ only those risk factors most strongly associated with the outcome were tested in the multivariable analysis (*P* < 0.1) and limited to a maximum of 4–5 parameters in the fully adjusted model. The population attributable fraction (PAF) was calculated using the formula:^27^,^28^

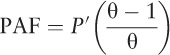
where *P′* = proportion of cases exposed and θ = the adjusted odds ratio (OR) of the association of M. tuberculosis infection in children with the risk factor.

Analyses were performed using Stata v. 13.1 for Mac (Stata Corporation, College Station, TX, USA).

### Ethics approval

The study was approved by the Malawi National Health Sciences Research Committee, Lilongwe, Malawi, and the London School of Hygiene & Tropical Medicine Ethics Committee, London, UK. Written informed consent was provided by a parent or guardian of each participating child.

## RESULTS

**Figure 1:**
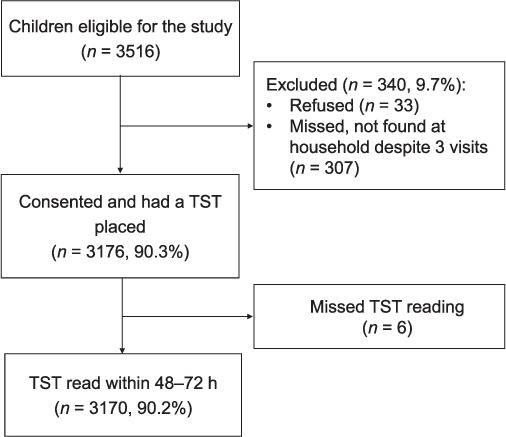
Flow diagram showing study participant flow at each stage from eligibility through to analysis, including non-participation. TST = tuberculin skin test.

A total of 3516 children aged 2–4 years were resident in the DSS and eligible to take part, 3170 (90.2%) of whom underwent a TST (read within 72 h) (Figure 1). Twenty-six children (0.8%) had lived in the same household as an adult with diagnosed infectious TB, and altogether 606 children (19.1%) had lived within 200 m of an adult with diagnosed infectious TB during their lifetime; 87 children (2.7%) were born to HIV-positive mothers, and altogether 470 (14.8%) children had lived in a household with at least one HIV-positive household member.

### Tuberculin data

**Figure 2:**
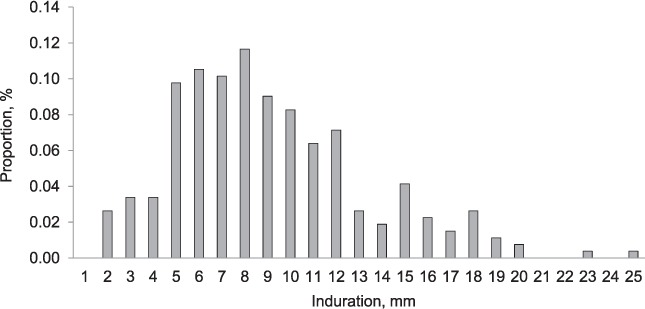
Observed frequency distribution of non-zero TST induration in children aged 2–4 years (*n* = 266). TST = tuberculin skin test.

An inter-rater reliability analysis on a sample of 215 TSTs of reading induration size to within 1 mm was found to be excellent (κ = 0.85, 95% conference interval [CI] 0.78–0.92), although this does not preclude digit bias; 91.6% of children aged 2–4 years, the majority (*n* = 2340, 81%) of whom had been BCG-vaccinated within the first year of life, had no induration in response to TST. The frequency distribution of non-zero indurations, shown in Figure 2, shows a bimodal distribution with modes at 8 mm and 15 mm, and some evidence of digit preference (at 5 mm, 8 mm and 15 mm).

**Figure 3:**
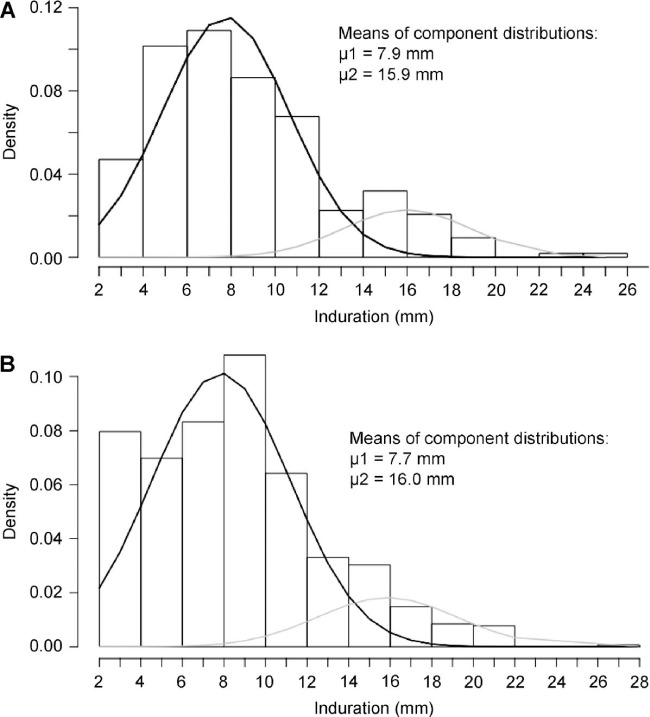
Mixture analysis of tuberculin data **A)** from 2012 in DSS (current study) and **B)** from 1980 to 1984 in Karonga District. DSS = demographic surveillance site.

Mixture analysis of non-zero induration data in this study (Figure 3A) generated a two-component distribution, with a mean at 7.9 mm representing sensitisation to environmental mycobacteria and/or BCG vaccination, and a mean of 15.9 mm representing M. tuberculosis infection. Mixture analysis of non-zero induration data from Karonga District in 1980–1984, restricted to those in the same age group with a BCG scar, revealed a strikingly similar distribution, with means at 7.7 mm and 16.0 mm (Figure 3B).

Of 2782 non-HIV-exposed children, 2555 (91.8%) had zero indurations, compared to 89.7% (78/87) of HIV-exposed children (χ^2^, *P* = 0.5). The median and interquartile range (IQR) of the non-zero indurations were similar in HIV-exposed (*n* = 9, median 8 mm, IQR 8–15) and non-HIV-exposed children (*n* = 227, median 8 mm, IQR 6–11).

### Prevalence and estimated annual risk of M. tuberculosis infection

Of the 3170 children, 35 had a TST induration of ⩾15 mm, giving an estimated M. tuberculosis infection prevalence of 1.1% (95%CI 0.8–1.6), adjusted for clustering by residential area. The mean age of children included in the analysis was 3.5 years, giving an estimated average annual risk of M. tuberculosis infection (ARTI) of 0.3% (95%CI 0.2–0.5).

### Risk factors associated with prevalent M. tuberculosis infection

Distance from the nearest known TB case during the child's lifetime, being resident in an area with an average smear-positive notification rate of >30/100 000 population/year (community M. tuberculosis exposure), being born to an HIV-positive mother, cohabiting with ⩾3 adult (age ⩾15 years) household members over the child's lifetime, dwelling score and age at BCG vaccination were associated with a positive TST on univariable analysis (Table 1). Adjusting for age and sex did not change the effect estimate for any of the risk factors examined. There was evidence of a dose-response effect of decreasing distance from the nearest known infectious TB case (*P* trend across categories = 0.02), with the highest ORs in those living within the same household, compared to children who lived >200 m from the nearest infectious case.

**Table 1:**
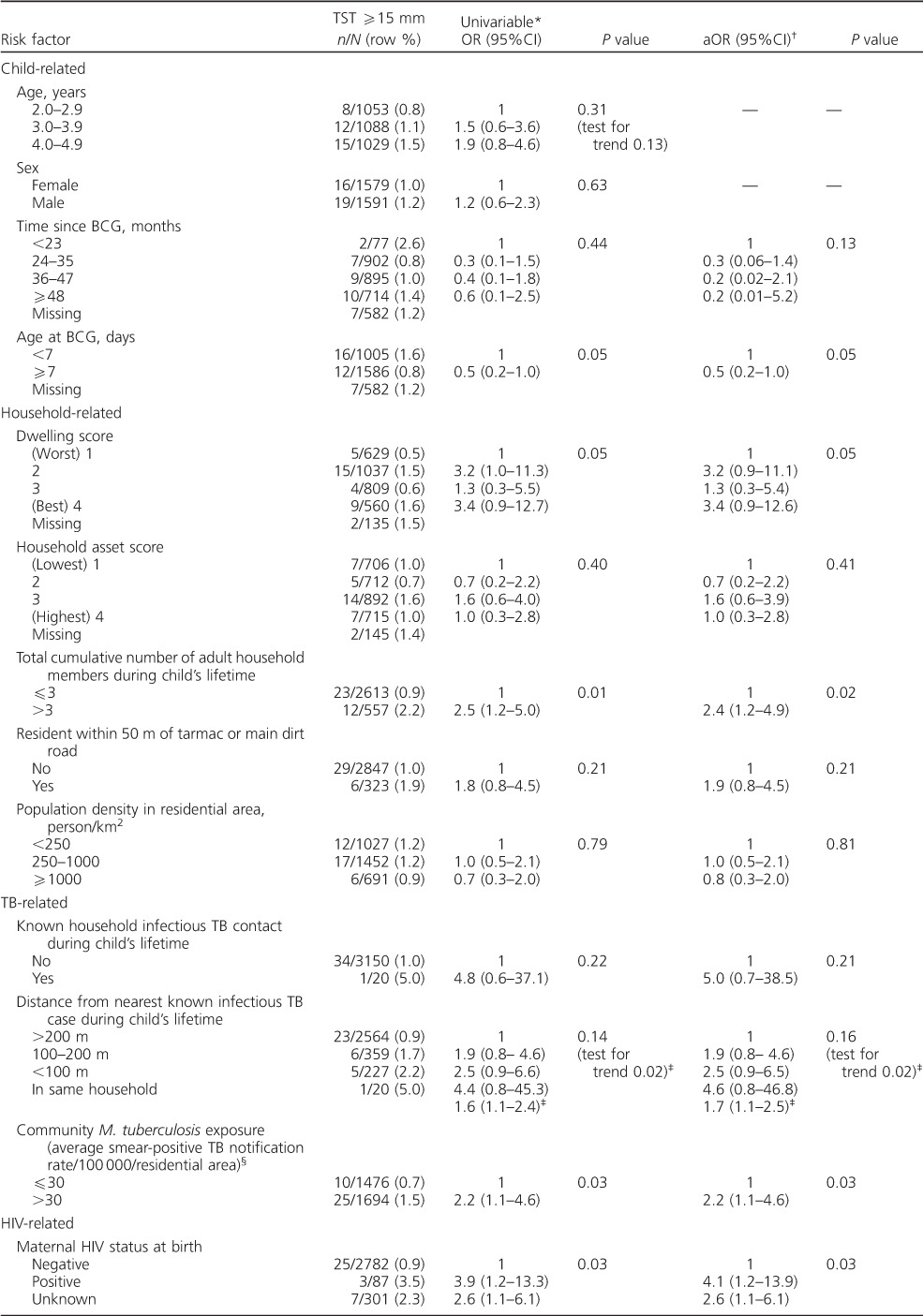
Risk factors associated with a positive TST (⩾15 mm) (*n* = 3170)

**Table 1:**
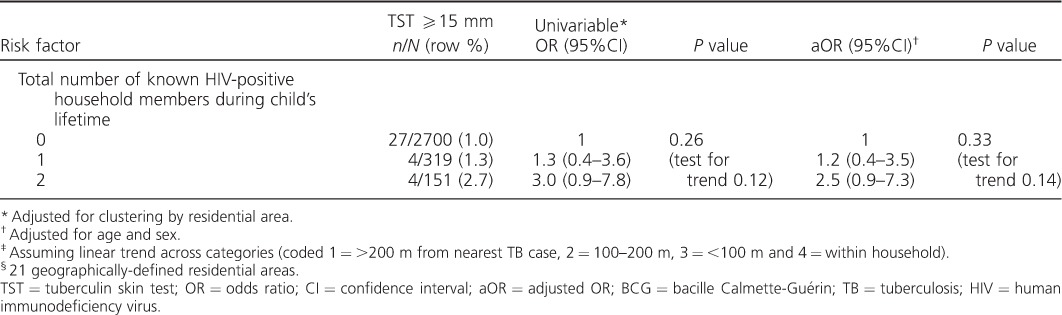
Risk factors associated with a positive TST (⩾15 mm) (*n* = 3170)

In a multivariable model, distance from the nearest known infectious TB case, cumulative number of adult household members and maternal HIV status at birth were the only risk factors that remained strongly associated with the risk of a positive TST (*P* < 0.05; Table 2). The adjusted ORs for these three risk factors were similar to the crude ORs.

**Table 2:**
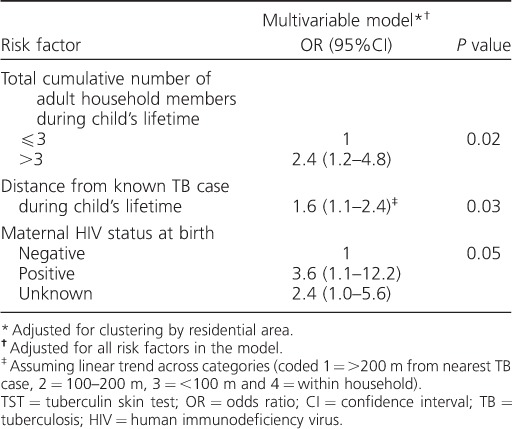
Multivariable analysis of risk factors associated with TST ⩾15 mm (*n* = 3170)

The odds of M. tuberculosis infection was two-fold higher in children living within 200 m of an infectious adult TB case (excluding household contact) compared to children living >200 m from an infectious adult TB case (aOR 2.1, 95%CI 1.0–4.5, *P* = 0.05, adjusted for HIV exposure status and number of adult household members). The odds of M. tuberculosis infection in children with known household contact was 3.6 times higher than among children with no known household contact (aOR 3.6, 95%CI 0.5–28.6, *P* = 0.3, adjusted for HIV exposure status and number of adult household members). The proportion of M. tuberculosis infection attributable to non-household contact with diagnosed infectious TB within 200 m (the PAF) was estimated at 17.0%, and the PAF for household contact was 2.3%.

## DISCUSSION

Despite the known lack of specificity of TST, tuberculin surveys remain a valuable epidemiological tool and provide important information on trends in tuberculous infection.^29^ These surveys are usually undertaken in school-aged children (6–11 years), and there is thus a paucity of data on the risk of recent M. tuberculosis infection as inferred by TST positivity in the very young.^30^,^31^ In our study, the prevalence of M. tuberculosis infection in children aged 2–4 years was approximately 1.1%, using a TST cut-off of 15 mm, yielding an estimated ARTI of 0.3% (95%CI 0.2–0.5).

Using the tuberculin data from Karonga District in 1980–1984, and restricting this study to a comparable population (aged 2–4 years with BCG scar) and the same methods, the estimated prevalence of M. tuberculosis infection was 2.3% (95%CI 1.8–2.8) and the ARTI was 0.7% (95%CI 0.5–0.8). Replication of surveys, with identical antigens and procedures, in the same district and segments of the population (as attempted in this study) is probably the most accurate way to assess changes in the risk of M. tuberculosis infection.^32^ Mixture analysis of tuberculin data from this study and from the earlier survey demonstrated strikingly similar two-component distributions, supporting the use of the 15 mm cut-off. The results suggest a reduction in the risk of infection in this age group over time, which is consistent with observed trends in incidence of smear-positive adult TB in the district.^17^ Our ARTI estimate of 0.3% may not reflect the ARTI in older children and adults in this setting, due to different social mixing patterns,^33^,^34^ although the determinants of the ARTI in this study population of very young children may in fact reflect the social mixing patterns and transmission dynamics of the mother or main carer.^35^

The association with neighbourhood TB was expected. A study in a township in Cape Town, South Africa, found a similar doubling in the odds of TST positivity (using TST ⩾10 mm) in children who lived on the same plot as an adult with smear-positive TB, compared to children with no plot-related exposure.^3^ In a setting with a low-to-moderate TB burden such as Karonga, the relative importance of household transmission might be expected to be greater than in Cape Town, where the very high TB incidence might make community transmission more important. It should be noted that despite the association with distance to the nearest infectious TB case, the PAF in our setting was very low, at 2.3% for household contact and 17.0% for neighbourhood (within 200 m), excluding household contact. This suggests that the maximum public health impact of treating all neighbourhood child contacts (aged <5 years) of known adult infectious TB cases with IPT in this population would not be substantial.

Children born to HIV-positive mothers had a 3.6-fold increased odds of M. tuberculosis infection, after adjusting for distance to known TB cases and the number of adult household members. An increased risk of M. tuberculosis infection in HIV-exposed, non-infected children compared to non-HIV-exposed children in early childhood was also seen in a Ugandan study, which used both TST and an interferon-gamma release assay to diagnose M. tuberculosis infection status.^36^ We did not know the HIV status of the children or the maternal M. tuberculosis infection status. It is unclear in our study whether the increased risk of M. tuberculosis infection seen in HIV-exposed children is due to increased susceptibility to M. tuberculosis infection following exposure and/or increased exposure, via either occult infectious TB within the household or unmeasured lifestyle factors associated with maternal HIV, such as increased attendance at health facilities.

Interestingly, no association between M. tuberculosis infection and population density was found. A plausible explanation may be that M. tuberculosis infections in young children in this community occur as a result of social mixing, which is unrelated to the population density of the area in which the child resides. There may be more undiagnosed infectious TB cases among females (the usual care givers of young children in rural Malawi), particularly in areas with lower population density, which are further from primary health care facilities than areas with the highest population density. A study is currently ongoing in the DSS to see if it is possible to identify the source of incident M. tuberculosis infection in children. Incident M. tuberculosis infection is identified by a repeat TST 1 year later in children with a previously negative TST. Household, neighbourhood and family contacts of children identified with incident M. tuberculosis infection are then screened for TB. It is hoped that this type of targeted case finding may identify a subset of undiagnosed cases that are responsible for onward M. tuberculosis transmission to the youngest members within the community.

The 1% of young M. tuberculosis-infected children in this population act as a sentinel for infectious TB in the community. In this setting, the majority of transmission events take place outside the household, either from unidentifiable casual contacts (with known or unknown TB) or from undiagnosed close contacts. This has major implications for strategies aiming to interrupt transmission. This study highlights the need to identify people with undiagnosed infectious TB in the community, the need to identify congregate transmission settings and the need to develop strategies to improve community infection control, irrespective of the presence of people with known TB. IPT for household contacts of smear-positive TB cases, although justified given the high risk to individual children, is predicted to have little effect on the population reservoir of infection, even with effective implementation in settings such as this.

## CONCLUSION

The majority of M. tuberculosis infections in children in our setting, which has a well-implemented TB programme, occur either from casual contact with infectious TB in the community or from undiagnosed infectious TB in close contacts. A better understanding of the loci of M. tuberculosis transmission to young children is needed to effectively target prevention interventions to mitigate future TB-related morbidity and mortality in children and ultimately improve longer-term TB control.

## References

[1] Bloch A B, Snider D E (1986). How much tuberculosis in children must we accept?. Am J Public Health.

[2] Rieder H L. (1999). Epidemiological basis of tuberculosis control.

[3] Middelkoop K, Bekker L G, Morrow C, Zwane E, Wood R. (2009). Childhood tuberculosis infection and disease: a spatial and temporal transmission analysis in a South African township. S Afr Med J.

[4] Marais B J, Obihara C C, Warren R M, Schaaf H S, Gie R P, Donald P R. (2005). The burden of childhood tuberculosis: a public health perspective. Int J Tuberc Lung Dis.

[5] Marais B J, Gie R P, Schaaf H S, Beyers N, Donald P R, Starke JR. (2006). Childhood pulmonary tuberculosis: old wisdom and new challenges. Am J Respir Crit Care Med.

[6] Diel R, Loddenkemper R, Zellweger J P (2013). Old ideas to innovate TB control: preventive treatment to achieve elimination. Eur Respir J 2013.

[7] Esmail H, Barry C E, Young D B, Wilkinson R J. (2014). The ongoing challenge of latent tuberculosis. Philos Trans R Soc Lond B Biol Sci.

[8] Morrison J, Pai M, Hopewell P C. (2008). Tuberculosis and latent tuberculosis infection in close contacts of people with pulmonary tuberculosis in low-income and middle-income countries: a systematic review and meta-analysis. Lancet Infect Dis.

[9] Verver S, Warren R M, Munch Z (2004). Proportion of tuberculosis transmission that takes place in households in a high-incidence area. Lancet.

[10] Crampin A C, Glynn J R, Traore H (2006). Tuberculosis transmission attributable to close contacts and HIV status, Malawi. Emerg Infect Dis.

[11] Buu T N, van Soolingen D, Huyen M N (2010). Tuberculosis acquired outside of households, rural Vietnam. Emerg Infect Dis.

[12] Schaaf H S, Michaelis I A, Richardson M (2003). Adult-to-child transmission of tuberculosis: household or community contact?. Int J Tuberc Lung Dis.

[13] Wood R, Johnstone-Robertson S, Uys P (2010). Tuberculosis transmission to young children in a South African community: modeling household and community infection risks. Clin Infect Dis.

[14] Madico G, Gilman R H, Checkley W (1995). Community infection ratio as an indicator for tuberculosis control. Lancet.

[15] Zelner J L, Murray M B, Becerra M C (2014). Age-specific risks of tuberculosis infection from household and community exposures and opportunities for interventions in a high-burden setting. Am J Epidemiol.

[16] Middelkoop K, Bekker L G, Morrow C, Lee N, Wood R. (2014). Decreasing household contribution to TB transmission with age: a retrospective geographic analysis of young people in a South African township. BMC Infect Dis.

[17] Mboma S M, Houben R M, Glynn J R (2013). Control of (multi)drug resistance and tuberculosis incidence over 23 years in the context of a well-supported tuberculosis programme in rural Malawi. PLOS ONE.

[18] Jahn A, Crampin A, Glynn J, Mwinuka V (2007). Evaluation of a village-informant driven demographic surveillance system. Demographic Res.

[19] Crampin A C, Dube A, Mboma S (2012). Profile: the Karonga Health and Demographic Surveillance System. Int J Epidemiol.

[20] Boccia D, Hargreaves J, Ayles H, Fielding K, Simwinga M, Godfrey-Faussett P. (2009). Tuberculosis infection in Zambia: the association with relative wealth. Am J Trop Med Hyg.

[21] Odone A, Crampin A C, Mwinuka V (2013). Association between socioeconomic position and tuberculosis in a large population-based study in rural Malawi. PLOS ONE.

[22] Dye C, Scheele S, Dolin P, Pathania V, Raviglione M C. (1999). Consensus statement. Global burden of tuberculosis: estimated incidence, prevalence, and mortality by country. WHO Global Surveillance and Monitoring Project. JAMA.

[23] Arnadottir T, Rieder H L, Trébucq A, Waaler H T. (1996). Guidelines for conducting tuberculin skin test surveys in high prevalence countries. Tubercle Lung Dis.

[24] Davies G R, Fine P E, Vynnycky E. (2006). Mixture analysis of tuberculin survey data from northern Malawi and critique of the method. Int J Tuberc Lung Dis.

[25] Benaglia T, Chauveau D, Hunter D R, Young D. (2009). mixtools: an R package for analyzing finite mixture models. J Stat Software.

[26] Peduzzi P, Concato J, Kemper E, Holford T R, Feinstein A R. (1996). A simulation study of the number of events per variable in logistic regression analysis. J Clin Epidemiol.

[27] Greenland S, Drescher K. (1993). Maximum likelihood estimation of the attributable fraction from logistic models. Biometrics.

[28] Rockhill B, Newman B, Weinberg C. (1998). Use and misuse of population attributable fractions. Am J Public Health.

[29] Borgdorff M. (2002). Annual risk of infection—time for an update?. Bull World Health Organ.

[30] Joos T J, Miller W C, Murdoch D M. (2006). Tuberculin reactivity in bacille Calmette-Guerin vaccinated populations: a compilation of international data. Int J Tuberc Lung Dis.

[31] Reid J K, Ward H, Marciniuk D, Hudson S, Smith P, Hoeppner V. (2007). The effect of neonatal bacille Calmette-Guerin vaccination on purified protein derivative skin test results in Canadian aboriginal children. Chest.

[32] Fine P E, Bruce J, Ponnighaus J M, Nkhosa P, Harawa A, Vynnycky E. (1999). Tuberculin sensitivity: conversions and reversions in a rural African population. Int J Tuberc Lung Dis.

[33] Buu T N, Quy H T, Qui N C, Lan N T, Sy D N, Cobelens F G. (2010). Decrease in risk of tuberculosis infection despite increase in tuberculosis among young adults in urban Viet Nam. Int J Tuberc Lung Dis.

[34] Wood R, Liang H, Wu H (2010). Changing prevalence of tuberculosis infection with increasing age in high-burden townships in South Africa. Int J Tuberc Lung Dis.

[35] Mossong J, Hens N, Jit M (2008). Social contacts and mixing patterns relevant to the spread of infectious diseases. PLOS MED.

[36] Marquez C, Chamie G, Achan J Tuberculosis infection in early childhood in Uganda and the influence of HIV exposure. http://www.croiwebcasts.org/console/player/22177?mediaType=slidevideo&.

